# Endoscopic Ultrasound-Guided Radiofrequency Ablation (EUS-RFA) for Pancreatic Adenocarcinoma: A Review

**DOI:** 10.7759/cureus.70691

**Published:** 2024-10-02

**Authors:** Antonio Costanzo, Emanuele Fulco, Michele Marini, Andrea Rigamonti, Lorenzo Vescovi, Antonio Floridi, Antonella Nisi, Elisa Pelfini, Elia Armellini, Antonio Piazzini Albani

**Affiliations:** 1 General and Emergency Surgery, Azienda Socio Sanitaria Territoriale (ASST) Bergamo Est, Seriate, ITA; 2 Post-Graduate School of General Surgery, Università degli Studi di Milano (La Statale), Milan, ITA; 3 Gastroenterology and Endoscopy, Azienda Socio Sanitaria Territoriale (ASST) Bergamo Est, Seriate, ITA

**Keywords:** radiotherapy, chemotherapy, radiofrequency ablation, endoscopic ultrasound, pancreatic adenocarcinoma

## Abstract

Pancreatic ductal adenocarcinoma (PDAC) is still one of the deadliest neoplasms in the world. Although various advancements in the treatment and management of this disease have been made, no significant overall survival benefit has been achieved. Endoscopic ultrasound-guided radiofrequency ablation (EUS-RFA) has been proposed as a treatment for patients who are unfit for surgery or with inoperable PDAC. We conducted a literature review of the PubMed and Embase databases to identify and analyze studies on the use of EUS-RFA in inoperable PDAC. Eleven studies with a total of 122 patients were analyzed to assess the population characteristics, feasibility and safety of the procedure, and overall survival of the population. Technical success was achieved in 95.1% of cases, and no intraoperative complications were reported. The most common early complication reported was abdominal pain (21 out of 122 patients) with a total early complication rate of 29.6%, and none of these complications affected hospital stays or post-procedure recovery. Late complications were reported in four patients (3.2%). Post-procedure cytoreduction was achieved in all patients, although disease progression was reported in 119 of 122 patients. The overall survival rate did not differ from that reported in the literature. We found that EUS-RFA could be a valid palliative option for inoperable patients, a bridge for surgery reducing the size of the tumor and its vascular relationship, or a first-line therapy in a subset of selected patients. Larger cohort and prospective studies should be conducted to establish guidelines for this procedure.

## Introduction and background

Despite advancements in both diagnostic and therapeutic interventions, pancreatic cancer remains one of the main causes of cancer-related deaths in the United States. In 2020, as estimated by the Global Cancer Observatory, the total number of diagnoses was approximately 496,000 worldwide with a mortality prevalence of 466,000. These numbers are estimated to double by 2040 [1].

Pancreatic cancer has a poor prognosis with a five-year survival rate of approximately 5%. Surgical resection is the only potential curative option for pancreatic cancer, and only 15-20% of patients are candidates for surgical resection at diagnosis because most of them have locally advanced cancer or metastatic disease. Pancreatic adenocarcinoma is defined as inoperable in three situations: peritoneal or distant metastases, presence of vascular involvement greater than 180 degrees of the superior mesenteric artery and/or celiac artery, and patients unfit for surgery for their comorbidities [2].

A multimodal approach is required in the management of pancreatic cancer. It involves systemic chemotherapy (adjuvant/neoadjuvant), radiotherapy, surgical resection, and endoscopic treatment [3].

For patients with unresectable pancreatic cancer, palliation with chemoradiation therapy and endoscopic radiofrequency ablation are used to improve the quality of life, even if these treatments rarely change the prognosis of patients. Recently, other treatments have been described, including targeted therapies, immunotherapies, angiogenesis inhibitors, and epidermal growth factor receptor inhibitors [4].

Radiofrequency ablation (RFA) is a well-known procedure that has been used effectively for the treatment of solid tumors, such as hepatocellular cancer [5]. RFA delivers thermal energy to the tumor through a needle electrode, which causes tumor cytoreduction through different mechanisms, such as coagulative necrosis, protein denaturation, and activation of the immune system in the tumor microenvironment [6].

RFA of pancreatic tumors can be performed using different modalities: intraoperative, ultrasound (US) or computed tomography (CT)-guided percutaneous RFA, and endoscopic ultrasound-guided RFA (EUS-RFA). Moreover, RFA with stent placement could be used in cases of biliary or pancreatic duct obstruction caused by unresectable tumors. Stent patency is prolonged through the reduction of tumor volume and the immunomodulatory effects on tumor regrowth [7].

EUS-RFA is a minimally invasive approach that involves the passage of an electrode needle through an endoscopic and ultrasonographic probe into the tumor [8]. There are no guidelines for EUS-RFA in the treatment of pancreatic adenocarcinoma, and the current application is based on the clinical experience of high-volume centers for pancreatic pathology. We conducted a literature review regarding EUS-RFA, focusing on inoperable pancreatic adenocarcinoma, to summarize and analyze the data available on this procedure.

## Review

Methods

In our study, we included articles published in English that analyzed patients treated with EUS-RFA for inoperable adenocarcinoma of the pancreas from 2007 to 2023. All studies were found by searching PubMed and Embase databases.

Inclusion Criteria

Criteria for inclusion were as follows: case reports and prospective, retrospective, and case-control studies on human patients with pancreatic adenocarcinoma who underwent EUS-RFA.

Exclusion Criteria

Criteria for exclusion were as follows: non-English articles, reviews, unspecified histology of the lesions, non-pancreatic adenocarcinoma, studies on animals, and other types of ablations.

Keywords

The keywords used were "Endoscopic Radio Frequency Ablation," "EUS-RFA," "Pancreatic adenocarcinoma," "locally advanced pancreatic ductal adenocarcinoma (LAPDAC)," and "inoperable pancreatic ductal adenocarcinoma (PDAC)."

Following an initial search that yielded 223 results, we screened these articles by reviewing titles, authors, and abstracts. Two hundred and one studies were excluded because of 130 duplicates and 71 irrelevant publications (treatment of other types of cancer or incomplete information). Upon reviewing the full articles, we excluded 11 articles that did not meet the inclusion criteria: three reviews, five studies on animals, and three studies for unspecified histology. The final analysis was carried out on a total of 11 articles.

Review

Our review primarily focused on analyzing the feasibility, defined as technical success, and safety in which we analyzed early and late complications of the procedure. We did not find any information regarding the use of the transgastric or duodenal approach for delivering therapy. Finally, we examined the outcomes of the follow-up reported in the studies, including tumor volume reduction, disease progression, and overall survival. Technical and clinical characteristics of the studies are reported in Table 1. 

**Table 1:** Clinical features and outcomes *Comparative study that compares inoperable adenocarcinomas treated with only CHT and with CHT plus EUS-RFA EUS-RFA: endoscopy ultrasound-guided radiofrequency ablation; HTP: HybridTherm Probe; N/A: not available; IPMN: intraductal papillary mucinous neoplasm; CHT: chemotherapy; OS: overall survival

	Location of pancreatic lesions, n						Response		
Title	Head/neck	Body/tail	N/A	Diameter of lesion, mean (range), mm	Technical success %	Treatment (needle caliber, sessions/lesions)	Complications		Median OS, months (median follow-up)
							Early (until seven days from the procedure)	Late (after seven days and within three months from the procedure)	
Arcidiacono et al. (2012) [9]	7	8	1	35.7 (25-34)	72.8%	EUS-RFA (HTP) (22G, 25G, 16/16)	Three cases of abdominal pain, one case of duodenal bleeding	Two cases of jaundice, one case of duodenal stenosis, one case of cystic fluid collection	6 (6)
Song et al. (2016) [10]	4	2	0	38 (30-90)	100%	EUS-RFA (18G 8/6)	Two cases of abdominal pain	Not reported	(4)
Crinò et al. (2018) [11]	3	3	2	36 (22-67)	100%	EUS-RFA (18G, 12/8)	Three cases of mild abdominal pain	Not reported	(6.5)
Scopelliti et al. (2018) [12]	4	6	0	(35x25 to 75x64)	100%	EUS-RFA (18G, 14/10)	Two cases of ascites, two cases of peri-pancreatic effusion	Not reported	(1)
Thosani et al. (2022) [13]	6 (one ampullary adenocarcinoma)	4	0	39.2 (12x14 to 68x50)	100%	EUS-RFA (19G or 22G, 22/10)	Mild abdominal pain in 55% of cases (12/22)	None	13.4
Ramireddy et al. (2019) [14]	-	-	-	-	100%	EUS-RFA (19G, 18/10)	None	None	5.5
Guider et al. (2018) [15]	5	5	0	27	100%	EUS-RFA (19G, 22G, X/10)	Mild abdominal pain in one case	None	(4.5)
Ligresti et al. (2019) [16]	1	-	-	15	100%	EUS-RFA (19G)	None	None	12
Robles-Medranda et al.(2022) [17]	24 adenocarcinomas, localization not specified (15/26 T4N0) (11/26 M1)			-	100%	EUS-RFA (19G)	None	Not reported	7
Testoni et al.* (2022) [18]	8	5	0	42.3 (±13.9)	100%	EUS-HTP (electroporation)	None	None	7
Tiankanon et al.* (2019) [19]	13 adenocarcinomas, not specified; one malignant IPMN, not specified			59.21 (±18.01)	100%	EUS-RFA	Three infections (site nonspecified), one mild pancreatitis, one bleeding at the puncture site	None	6 months OS=70%

We included a total of 122 patients in our study with inoperable PDAC. All patients underwent neoadjuvant chemotherapy and/or radiotherapy and were deemed inoperable due to locally advanced or distant disease, comorbidities, or advanced age. The male-to-female ratio for 75 patients was 0.95/1.05; in 47 cases, it wasn't specified. Patients varied in age with a range from 35 to 83 years old. The average age ranged from 61.9 years to 67.7 years.

Tumors were localized as follows: 38 out of 122 patients (31.1%) had a head/neck localization, 33 (27.1%) were localized in the body/tail region, and in 51 patients (41.8%), the tumor's location was not specified.

The mean maximum diameter of the tumors calculated for eight out of 11 studies was 40.04 mm [9-19]. 

General characteristics of the patients and studies are listed in Table 2.

**Table 2:** General overview of the studies and population HTP: HybridTherm Probe

Title	Year	Study type	Study interval	Number of patients, n (male, n)	Mean age (range)
Arcidiacono et al. [9]	2012	Prospective	September 2009-May 2011	16 (9)	61.9 (35-84)
Song et al. [10]	2015	Prospective	February 2013-March 2014	6 (1)	62 (43-73)
Crinò et al. [11]	2018	Prospective	November 2016-July 2017	8 (5)	67.7 (48-85)
Scopelliti et al. [12]	2018	Prospective	February 2016-October 2016	10 (3)	62 (50-74)
Thosani et al. [13]	2022	Prospective	October 2016-March 2018	10 (7)	62.3 (56.5-68)
Ramireddy et al. [14]	2019	Retrospective	2015-2018	10 (?)	-
Guider et al. [15]	2018	Prospective case-control	-	10 (7)	62.5 (59-66)
Ligresti et al. [16]	2018	Case report	-	1 (M)	83
Robles-Medranda et al. [17]	2022	Retrospective	October 2019-May 2021	24 (?)	-
Testoni et al. [18]	2022	Retrospective case-control (EUS-HTP)	2010-2016	13 (?)	64.8 (57-71)
Tiankanon et al. [19]	2019	Prospective case-control	July 2017-August 2018	14 (4)	66.53 (56.1-76.1)

Technical success is defined as the completion of the endoscopic procedure. Technical success was achieved in 95.1% of the cases, with 100% success reported in 10 studies and 72.8% success in the study by Arcidiacono et al. [9], which described six cases with stiffness of the gastrointestinal wall or tumor as the cause of failure. No major intraprocedural complications were reported.

Regarding safety, we analyzed the complications and divided them into early and late. Early complications were defined as those that arose until seven days from the procedure, and late complications were reported after seven days and within three months from the procedure. Some authors of reviewed studies report the use of rectal indomethacin as required by gastroenterological protocols; other authors do not report this data. There are no information regarding the use of prophylactic antibiotics.

The total early adverse events were 36 (29.6%); the most common early complication reported was mild abdominal pain in 21 out of 122 patients, which was treated conservatively. Other early complications reported were six cases of mild pancreatitis, two cases of bleeding at the puncture site, two cases of peri-pancreatic effusion, two cases of ascites, and three cases of infection (site not specified); these adverse events were also treated conservatively.

Late complications were reported in four patients (3.2%): two cases of jaundice, one case of duodenal stenosis, and one case of cystic fluid collection between the hepatic left lobe and the pancreas.

In all studies and in all patients, tumor cytoreduction was achieved after the procedure (100%). However, disease progression was reported in 119 out of 122 patients (97.5%).

Data on the median overall survival were extrapolated from 81 out of 122 patients. The median overall survival was eight months. The overall survival at 12 months was 33.3%, which declined to just 2.4% at five years.

There are no established guidelines for EUS-RFA in the treatment of pancreatic adenocarcinoma. All data in the literature derive from small studies limited to small cohorts of patients that have shown the safety and feasibility of EUS-RFA.

Our work is a literature review of 11 studies with a total of 122 patients, with an average age range of 61.9-67.7 years. Tumor locations were in the head/neck region in 31.1% of cases and in the body/tail region in 27.1% of cases, and in 41.8% of cases, it was not specified. Tumor localization, combined with the technical success rate calculated at about 95.1%, suggests the feasibility of the procedure regardless of tumor location.

The safety of the procedure was reviewed by taking into account early and late complications. Early complications were reported for 36 patients (29.6%): the most common complication was mild abdominal pain, treated with conservative therapy. Other early complications reported were as follows: six cases of mild pancreatitis, two cases of bleeding at the puncture site, two cases of peri-pancreatic effusion, two cases of ascites, and three cases of infection (site not specified). None of these events significantly impacted the overall hospital stay or post-procedure recovery.

Late complications were reported in four out of 122 patients (3.2%): two cases of jaundice, where both patients required endoscopic retrograde cholangiopancreatography (ERCP) and biliary stent positioning with full resolution, one case of duodenal stenosis treated conservatively, and one case of cystic fluid collection between the hepatic left lobe and the pancreas, treated conservatively.

Zhang et al. described a clinical success rate of EUS-RFA of 83.5% with an adverse events rate of 32.2% treated in the majority conservatively [20]. Alvarez-Sanchez and Napoléon reported a review of 42 patients who underwent EUS-RFA for different pancreatic lesions: unresectable pancreatic cancer in 28 patients, neuroendocrine tumors in seven, mucinous cysts in four, and microcystic adenoma in one. No procedure-related mortality was reported; mild early complications were observed in 25% of the patients, and only one case of mild acute pancreatitis occurred [21]. Other common adverse events described in the literature are pancreatic fistulas, gastrointestinal hemorrhage, sepsis, portal vein thrombosis, and duodenal or bile duct injuries [8].

Our data suggests that EUS-RFA is a safe procedure, with a very low risk of severe complications and mortality. Most complications are treated conservatively or with an endoscopic procedure.

The recommended thermal energy ranges from 60°C to 100°C because a temperature >100°C may result in a higher risk of adverse events due to a particular position of the pancreas and its fragile structure. In addition, several anatomical challenges may hamper the use of RFA for pancreatic cancer treatment. These include the retroperitoneal location of the pancreas; its close relation to the duodenum, stomach, transverse colon, and portal vein; and the involvement of the bile duct. During energy delivery, a circular area is spared at the tumor margins to reduce the risk of damage to surrounding vital structures. Moreover, complete ablation of the entire tumor may not be feasible in cases of retroperitoneal extension and vascular invasion [22].

Wu et al. recommended that the distance between the RFA site and major peri-pancreatic vessels should be >5 mm and the recommended safe temperature for RFA is 90°C [23].

Fegrachi et al. recommended a 10 mm distance from the duodenum and 15 mm from the portal and mesenteric vessels, as well as continuous cooling of the duodenum using 100 ml/min saline at 5°C to reduce the risk of duodenal injuries [24].

All studies described a tumor volume reduction after the procedure, but long-term tumor cytoreduction was not maintained.

Kongkam et al. described a case-control comparative study that compared 14 patients (group A) treated with EUS-RFA and systemic treatment versus 14 patients (group B) treated with systemic therapy only. They reported a significant reduction in analgesic use for group A, a significant increase in tumor diameter for group B, but no significant difference in survival at six months.

Disease progression was reported in 119 out of 122 patients (97.5%) [15]. Ligresti et al. and Thosani et al. reported that three patients who underwent surgery showed disease regression at the six-month follow-up. All the studies had limited post-procedure follow-up, mainly due to disease progression or study design [16,25].

The overall survival was 67.8% at six months, 33.6% at 12 months, and 2.4% at five years. These results are similar to those reported in the literature [2].

Thosani et al. reported that two patients were still alive at the five-year follow-up. One of the two patients (alive at 81-month follow-up) underwent robotic-assisted pancreato-duodenectomy, which resulted in a definitive histological diagnosis of ampullary adenocarcinoma [13]. The other one underwent laparoscopic duodeno-pancreatectomy for pancreatic adenocarcinoma and was still alive at the 61-month follow-up. Both had locally advanced diseases which were initially inoperable for vascular involvement.

Ligresti et al. reported a case of an 83-year-old man with a first-stage adenocarcinoma. He was deemed unfit for surgery due to his cardiovascular comorbidities and was still alive at the 12-month follow-up after EUS-RFA of a 15 mm neck pancreatic adenocarcinoma [16].

Discussion

Our review concerns the EUS-RFA treatment of pancreatic adenocarcinoma unlike other reviews in the literature which include all types of pancreatic neoplasms treated with this technique. The limitations of our study are related to the fact that these are studies with limited numbers of patients; this is linked to the fact that only a very limited and selected number of patients undergo this therapy given the complexity of pancreatic cancer.

Radical surgery with R0 margins remains, at the time of writing, the only curative option for LAPDAC; however, EUS-RFA could be used as a bridge therapy to surgery alone or in association with chemotherapy. Moreover, EUS-RFA could be used for palliative purposes and to reduce the dose of analgesic.

The use of RFA combined with chemotherapy could increase the rate of tumor cytoreduction through direct thermo-necrosis and tumor poration, which raises the penetration of chemotherapy in the tumor tissue. Tumor ablation also seems to improve the immune system response by enhancing tumor recognition through the diffusion of tumor antigens [26]. 

## Conclusions

EUS-RFA for PDAC could be a valid approach for a subset of strictly selected patients, as it is a relatively safe modality and a potential adjuvant to chemotherapy in the multidisciplinary management of unresectable pancreatic cancer. The current use of EUS-RFA in pancreatic cancer is based on individual experience and expertise in some centers using their own protocols. Guidelines for EUS-RFA do not exist in current practice, mainly due to the lack of sufficient data. Further large cohorts and standardized, randomized trials are required to achieve a better understanding of the efficacy of the procedure and to eventually establish guidelines.
